# A Practical Solution for 77 K Fluorescence Measurements Based on LED Excitation and CCD Array Detector

**DOI:** 10.1371/journal.pone.0132258

**Published:** 2015-07-15

**Authors:** Jacob Lamb, Kristin Forfang, Martin Hohmann-Marriott

**Affiliations:** Department of Biotechnology & PhotoSynLab, Norwegian University of Science and Technology, Trondheim, Norway; University of Hyderabad, INDIA

## Abstract

The fluorescence emission spectrum of photosynthetic microorganisms at liquid nitrogen temperature (77 K) provides important insights into the organization of the photosynthetic machinery of bacteria and eukaryotes, which cannot be observed at room temperature. Conventionally, to obtain such spectra, a large and costly table-top fluorometer is required. Recently portable, reliable, and largely maintenance-free instruments have become available that can be utilized to accomplish a wide variety of spectroscopy-based measurements in photosynthesis research. In this report, we show how to build such an instrument in order to record 77K fluorescence spectra. This instrument consists of a low power monochromatic light-emitting diode (LED), and a portable CCD array based spectrometer. The optical components are coupled together using a fiber optic cable, and a custom made housing that also supports a dewar flask. We demonstrate that this instrument facilitates the reliable determination of chlorophyll fluorescence emission spectra for the cyanobacterium *Synechocystis* sp. PCC 6803, and the green alga *Chlamydomonas reinhardtii*.

## Introduction

### 77 K fluorescence and photosynthesis

Analytical photosynthetic research relies on many photometric analysis methods. One such assay acquires the chlorophyll fluorescence emission spectrum at the temperature of 77 K (-196.15°C). Fluorescence measurements at 77 K are used to characterize the state of the photosynthetic machinery. At this temperature, photosynthetic reactions, with the exception of light harvesting and primary photochemistry, are inhibited. Analysis of 77K fluorescence spectra allows near-instantaneous assessment of the presence of photosystems and light harvesting systems and their interactions.

### 77 K fluorescence measuring techniques

Excitation of pigments (chlorophylls, carotenoids and phycobilins) that are part of the photosynthetic machinery causes a chlorophyll fluorescence emission that is nested in the orange to red light spectrum. As captured excitation is channeled between pigments, an emission spectrum at 77K does not represent the summed emission of all pigments, but can be thought of as reflecting these channeling pathways. Channeling of excitation follows an energy gradient that transfers energy from pigments with higher energy (shorter wavelength) to pigments with lower energy (longer wavelength), with the reaction centers as the ultimate recipients of the excitation energy. As a consequence, pigments of a light harvesting system, which is efficiently coupled to a reaction center, will emit very little fluorescence. In contrast, pigments of an unconnected light harvesting system will exhibit a larger fluorescence emission that is dominated by longer wavelength pigments, which cannot transfer excitation energy to the reaction center.

The reaction centers of many cyanobacteria, red algae, green algae, and vascular plants exhibit three major fluorescence peaks (F685, F695, and F700–735) at 77K [1]. The two fluorescence peaks at shorter wavelengths (F685 and F695) are derived from PS II antenna pigments, while the longer wavelength fluorescence (F710–735) is emitted by PS I antenna pigments [1–10].

Peak wavelengths associated with the PSII subunits CP43 and CP47 (approximately 685 and 695 nm) are fairly constant across cyanobacteria, algae and plants. The fluorescence emission associated with PSI at around 720 nm was first discovered in *Chlorella pyrenoidosa* at 77 K [11]; however, further studies indicated that the major emission band of PS I is remarkably variable among different species [1], with peaks ranging from 710 nm to 730 nm.

Phycobilin-containing organisms possess 77K fluorescence with features that are different from green algae and plants. The phycobilins are pigments that are associated with phycobiliproteins, which function as light harvesting systems. The excitation energy harvested by phycobilins is also channeled to reaction centers. In many cyanobacteria, phycobilins are organized into phycobilisomes that are mostly associated with PSII, but associate with PSI under a variety of stress conditions. Exciting phycobilins in cyanobacteria therefore usually channels excitation energy to PSII. The terminal emitter of the phycobilisomes (Lcm) possesses a fluorescence signature that contributes to the CP43-derived 685 peak [12]. Using phycobilisome excitation light, the connectivity between phycobilisomes and the two photosystems can be determined.

### Instrumentation for 77 K chlorophyll fluorescence

Generally, 77 K chlorophyll fluorescence emission measurements utilize table-top fluorometers. Many of these instruments require a start-up time for the illumination system and a calibration period of the optical detection system before the instrument is ready for use. In addition, they can be very expensive, so a suitable fluorometer may not be available in every laboratory. Therefore, an economic alternative may be desirable, especially if such an instrument offers rapid and reliable data acquisition.

LEDs have been embraced as light sources of choice for stable monochromatic illumination, as LEDs only require low power and low voltages for their operation, develop little heat, and are small in size. Due to these characteristics LEDs have been used for many portable applications including instruments dedicated to photometric analysis as long ago as the 1970s [13]. More recently, with the availability of LEDs in a variety of emission wavelengths, LED-based analytical devices have been developed for a variety of analytical purposes including those with relevance to photosynthesis [14–15].

Instrumentation for fluorescence detection relies on different modes of detection. Traditional fluorometers analyze a fluorescence spectra by scanning over the wavelength range of interest using a single detector, usually a photomultiplier or avalanche photodiodes. In these traditional instruments, physical movement of optical parts during the scanning process that delivers light to the detector requires time, usually at a range of 0.1–1 s per nm. The recent development of systems using charge-coupled device (CCD) arrays has considerably reduced the complexity and cost of optical instruments, with the cost of CCD spectrometers being several fold less expensive than bulky bench-top instruments. In a spectrometer that utilizes a CCD array no moving components are required, as a fixed chromatic grating generates a wavelength spectrum that is projected onto the CCD array. These setups allow whole spectrum acquisition, whose time resolution is only limited by the time required to read out the CCD array and the desired quality of the data. Charge-coupled device array systems have been recently designed and used for fluorescence detection in real-time PCR machines [16], ELISA plate readers [17, 18] and toxicity detectors [19]. Also these devices have been used in room-temperature applications for spectrophotometer [20], chlorophyll fluorescence [21], and photosynthetic kinetics [22].

In this report, we describe the construction of an instrument for the acquisition of chlorophyll fluorescence spectra at 77K that utilizes a CCD array detection system and LEDs for excitation. Using 3D printing techniques, we constructed a holder that interfaces these components with optical filters and a dewar that holds the sample. We compare the performance parameters of this system with a traditional table-top fluorometer using the cyanobacterium *Synechocystis* sp. PCC 6803 (*Synechocystis* hereafter), and the green alga *Chlamydomonas reinhardtii* (*Chlamydomonas* hereafter) as examples.

## Materials and Methods

### Cell growth

Liquid cultures of *Synechocystis* and *Chlamydomonas* were established in 300 mL Erlenmeyer flasks that had been specifically modified as described in Eaton-Rye, 2011 [23], and provided with filtered aeration via small aquarium pumps. *Synechocystis* cultures were grown in BG-11 media [24], supplemented with 5 mM glucose under constant illumination (20 μE • m^−2^ • s^−1^), at 30°C. *Chlamydomonas* cultures were grown in TAP media [25] under constant illumination (10 μE • m^−2^ • s^−1^), at 22°C.

### Custom 77 K fluorometer

**Fig 1:**
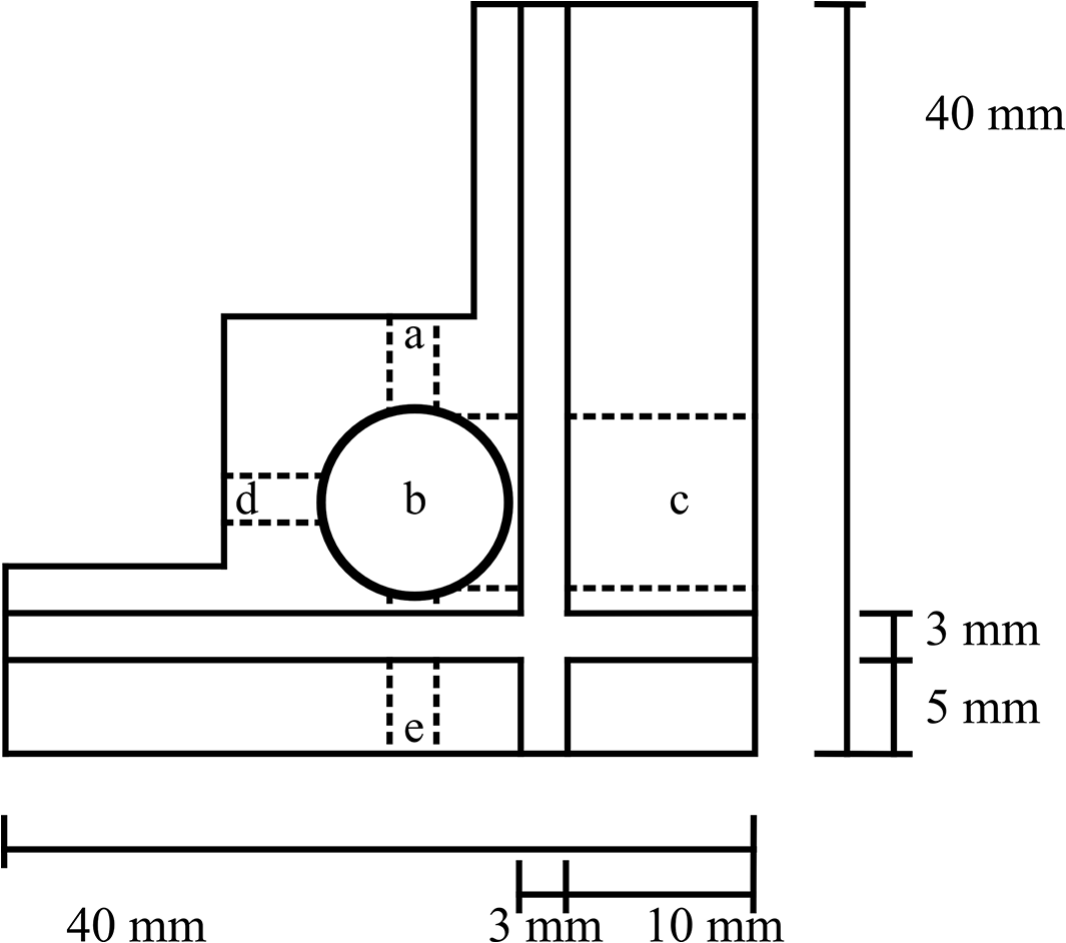
Apparatus housing schematic. The two 3 mm slots that span the housing are designed to fit standard 50 X 50 X 3 mm optical filters. The standard fluorescence LED input (a; 5 mm diameter) is for sample excitation, the dewar housing (b; 11 mm diameter) houses the dewar, and the optical fiber input (c; 10 mm diameter) positions the emission acquisition optical fiber close to the sample. The extra LED input (d; 5 mm diameter) is included for adaptation of absorption measurements, whereas the other extra LED input (e; 5 mm diameter) is an alternative fluorescence excitation input with the possibility of using an optical filter between the LED and the sample.

**Fig 2:**
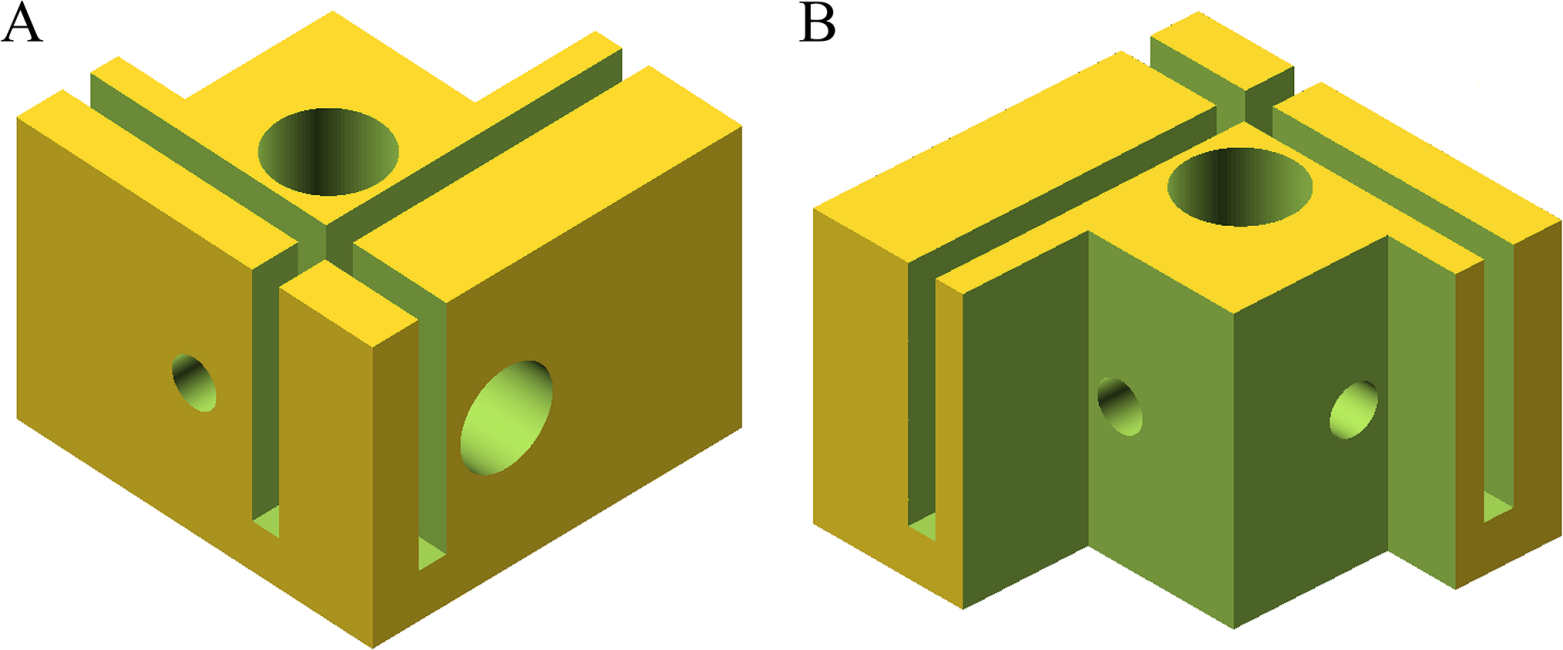
Apparatus housing 3-dimentional schematic. A 3-dimentional rendered schematic of both the front (a) and rear (b) sides of the housing designed to couple the LED and optical fiber with the dewar containing the sample.

LEDs were used to illuminate the sample. Excitation of chlorophylls was provided by a monochromatic LED with an emission centered at 435 nm (LED435-12-30, Roithner LaserTechnik). Additionally a monochromatic LED with emission at light centered at 572 nm was used for excitation of phycobilins (B5-433-20, Roithner LaserTechnik). The fluorescence of the sample is transferred to the CCD array spectrometer (JAZ-EL200, Ocean Optics) by an optical fiber located perpendicular to the direction of the light source. A 480 nm long-pass filter for the excitation of chlorophylls and a 580 nm long-pass filter for the excitation of phycobilins are inserted between the sample and the optical fiber to avoid the detection of short-wavelength LED light. The distance between the LED and the sample dewar is 1 mm; whereas the distance between the sample dewar and the fiber that is connected to the detector is 5 mm due to the space requirement of the optical filter. A schematic depicting the 2-dimensional layout is presented in Fig 1, while a detailed 3-dimentional view of the housing is presented in Fig 2.

#### Overview

The central component of the instrument is an Ocean Optics spectrometer (JAZ-EL200). The spectrometer is able to record whole spectrum from 200–850 nm, and communicates with a computer via a USB interface to display the data in quasi real time. With an entrance aperture of only 25 μm, the spectrometer possesses an optical resolution of 1.3 nm. Software for acquisition of spectra is available for Microsoft Windows, Mac OS, and Linux platforms. Data acquisition can be controlled by a computer via a USB cable. Additionally, the spectrometer can be used independently of a computer with an SD memory card for data storage.

#### Illumination

The LED light source is powered independently using a 5 V power supply. The 435 nm LED has a typical voltage of 3.4 V so a resistor was used to modulate the 5V power source (82 ohm resistor used, resulting in a 20.3 mW radiant flux, 74 nmol photons • s^-1^). The 572 nm LED has a typical voltage of 2.0 V so a resistor was used to modulate the 5V power source (150 ohm resistor used, resulting in a 20.0 mW radiant flux, 78 nmol photons • s^-1^).

### 77 K fluorescence

#### Sample preparation

*Synechocystis* and *Chlamydomonas* cells were harvested and resuspended in BG-11 media containing HEPES/NaOH (pH 7.5), and buffered TAP media (pH 7.0), respectively. Measurements of chlorophyll concentration and OD were made using a custom-built fluorometer/photometer [26–27]. Cells were then transferred into glass tubes (inner diameter 2 mm, outer diameter 5 mm) at a series of chlorophyll *a* concentrations and frozen in liquid nitrogen.

#### Conventional acquisition

Chlorophyll fluorescence spectra were obtained using a Horiba Jobin-Yvon Spex Fluorolog fluorometer with a 450 W Xenon lamp, and a thermoelectrically cooled R928P detector (240–850 nm). Excitation light was set to 435 nm (targeted at chlorophyll *a* absorption maxima) with a half-bandwidth of 10 nm (similar to the LEDs emission characteristics). Emission was detected perpendicular to the excitation light path. The emission spectrum was measured by scanning from 600–800 nm with a slit width of 1 nm (similar resolution to the custom made fluorometer).

## Results

Fluorescence emission spectra at 77 K are an important analytical parameter in photosynthetic research. To determine the emission characteristics of samples, we designed an instrument that utilizes a single LED as light source and measures the resulting fluorescence emission by a CCD array detector. In addition to demonstrating the performance of this instrument, this report contains technical details that should allow an operator with minimal background in electronics to construct and evaluate the fluorometer.

### Constructed Flurometer

**Fig 3:**
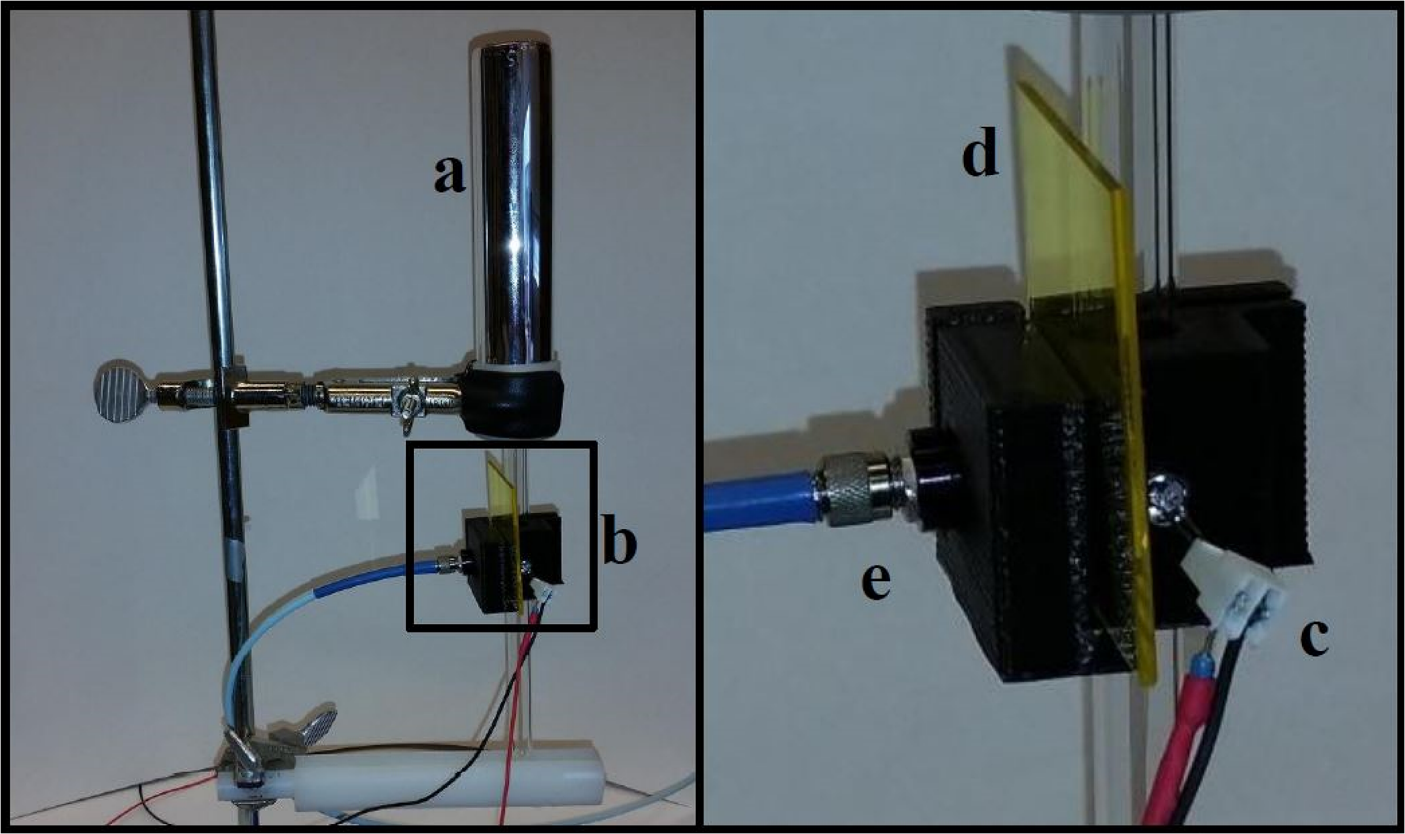
Fluorometer setup. Photographs of the custom setup on a standard utility clamp stand. The dewar (a) holds the liquid nitrogen, and the samples. The 3D-printed housing (b) provides fittings for the excitation LED (c), the long-pass filter (d) and optical fiber (e).

All components of the constructed fluorometer (excitation light source, fiber for the detection of fluorescence, optical filters, dewar) are fixed in place by a simple plastic holder (see Figs 1 & 2) so that the excitation LED and emission detecting fiber were orientated at a 90 degree angle. Fig 3 shows our constructed fluorometer.

The excitation system consists of LEDs chosen to excite chlorophylls (435nm) and phycobilins (580) nm. The half-band width of the used LEDs was determined to be 10 nm for both LEDs. The detection system consists of a CCD array detector that was coupled via an optical fiber to the sample holder. Optical long-pass filters were used in an effort to eliminate contribution of the LEDs to the fluorescence signal. The dewar, which holds the sample tubes, is inserted between the LED and emission fiber. During experiments, the entire setup was covered by a black cloth.

### Comparison of fluorometers

A series of experiments was performed to compare the performance parameters of the custom-made fluorometer to the performance of a traditional table-top fluorometer.

#### Cell Density

Cell density is an important parameter for 77K fluorescence measurements. Measurements at low cell concentrations are of advantage, as it is possible to acquire data without pre-concentration steps that could influence the physiological state of the sample. Furthermore, low cell concentrations avoid reabsorption of emitted fluorescence by the samples, which could distort the fluorescence emission spectra.

To compare the constructed instrument with the traditional fluorometer, commonly utilized acquisition parameters were applied. The integration time for the traditional fluorometer was set to 1 s • nm^-1^ resulting in an acquisition time of 200 sec for the spectrum from 600–800 nm.

**Fig 4:**
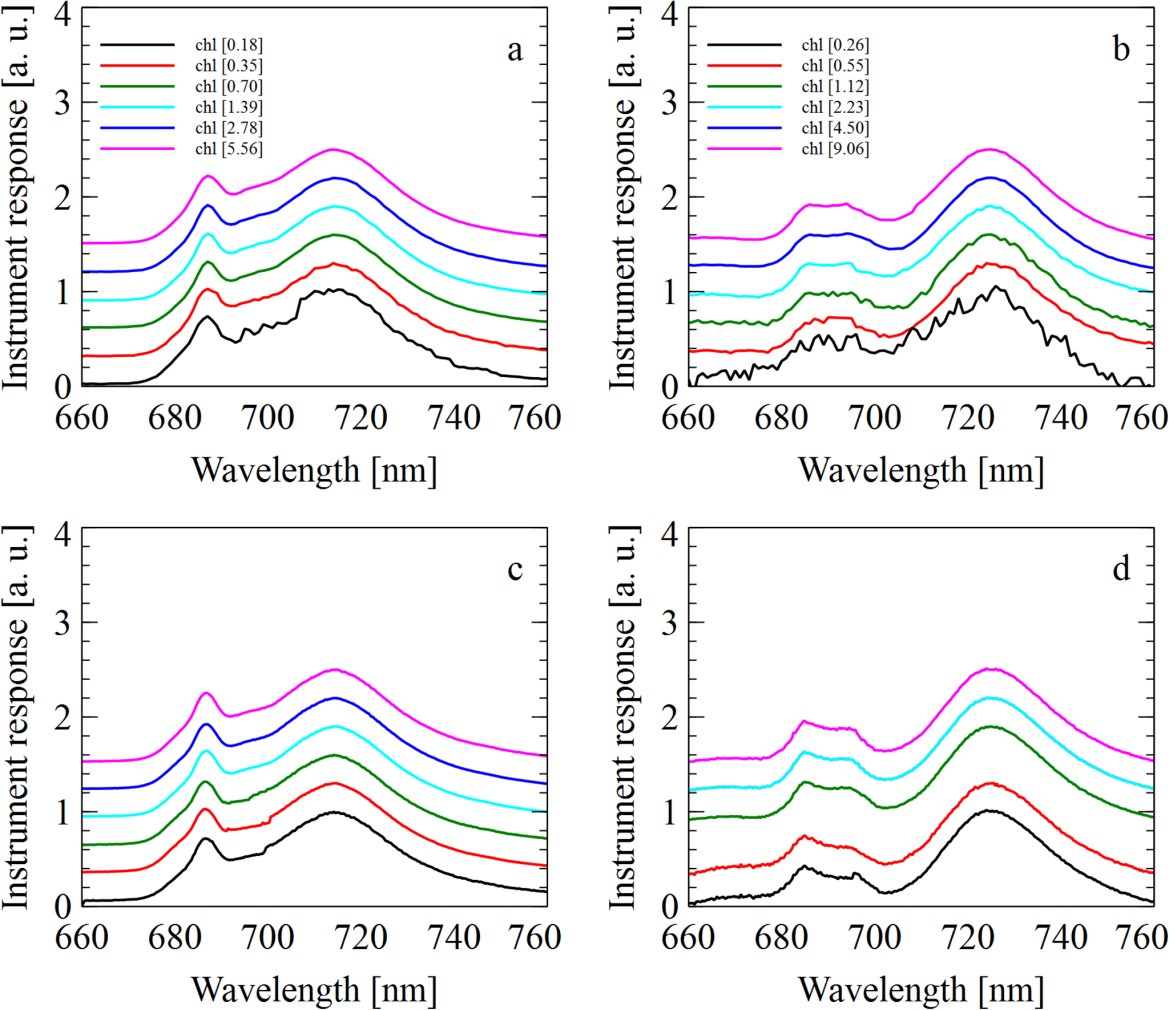
Off-set emission spectra. A series of 77 K fluorescence emission spectra were acquired with different cell chlorophyll concentrations for both *Chlamydomonas* (a, c; normalized to 715 nm) and *Synechocystis* (b, d; normalized to 725 nm). Spectra of increasing chlorophyll concentrations were normalized and shifted by 0.3 to assess noise of the spectra. The spectra were obtained from a traditional table top fluorometer (a, b), and the custom fluorometer (c, d). Chlorophyll concentrations are displayed in nmol • mL^-1^.

**Fig 5:**
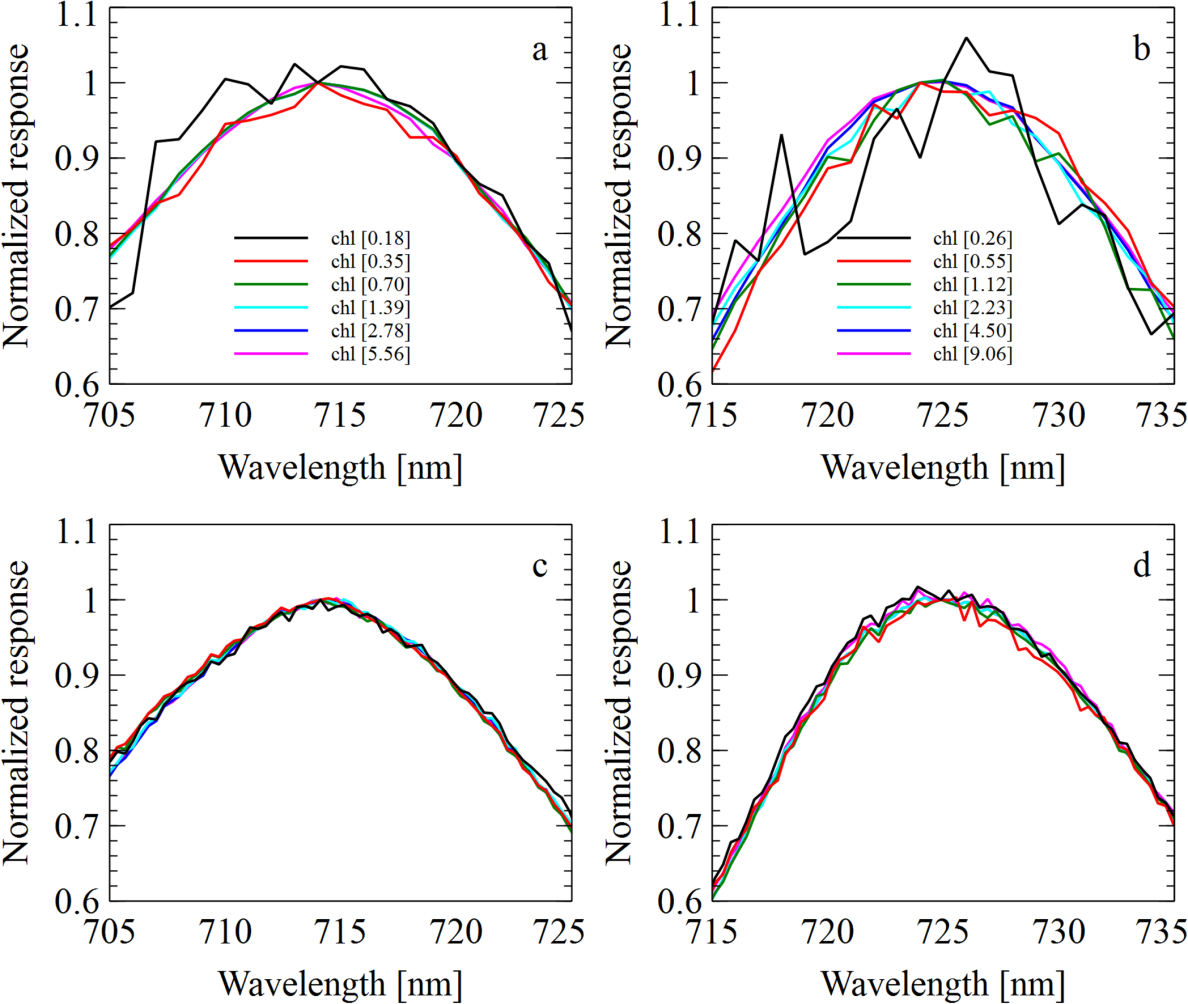
Fluorescence reabsorption comparison. A series of 77 K fluorescence emission spectra were acquired with different chlorophyll concentrations for both *Chlamydomonas* (a, c; normalized to 715 nm) and *Synechocystis* (b, d; normalized to 725 nm). The spectra were obtained from a traditional table-top fluorometer (a, b), and the custom fluorometer (c, d). Chlorophyll concentrations are displayed in nmol • mL^-1^.

Recorded spectra at different densities show that the custom-made 77K fluorometer is able to resolve fluorescence spectra down to a chlorophyll concentration of 0.18 nmol • mL^-1^ for *Synechocystis* and 0.26 nmol • mL^-1^ for *Chlamydomonas* (Fig 4). At chlorophyll concentrations as high as 9.06 nmol • mL^-1^ in *Synechocystis* samples and 5.56 nmol • mL^-1^ in *Chlamydomonas* samples, there is no observable shift of the 715 nm peaks of *Chlamydomonas* (Fig 5A and 5C), or the 725 nm peaks of *Synechocystis* (Fig 5B and 5D). This suggests there is insubstantial absorption of fluorescence emission in the samples at these chlorophyll concentrations. Table 1 shows the chlorophyll concentrations, OD and cell count values for all samples used.

**Table 1:** Sample standards of *Synechocystis* and *Chlamydomonas* used for calibration, optimization and comparison of 77 K instrumentation.

	*Synechocystis*			*Chlamydomonas*		
	OD_750_	chl *a* (nmol • mL^-1^)	Cells • mL^-1^ (× 10^7^)	OD_750_	chl *a* (nmol • mL^-1^)	Cells • mL^-1^ (× 10^6^)
**Standard 1**	0.10	0.26	0.41	0.10	0.18	0.51
**Standard 2**	0.20	0.55	0.81	0.20	0.35	0.99
**Standard 3**	0.40	1.12	1.63	0.40	0.70	2.02
**Standard 4**	0.80	2.23	3.23	0.80	1.39	4.05
**Standard 5**	1.60	4.50	6.51	1.60	2.78	8.18
**Standard 6**	3.20	9.06	13.05	3.20	5.56	16.23

#### Acquisition time

**Fig 6:**
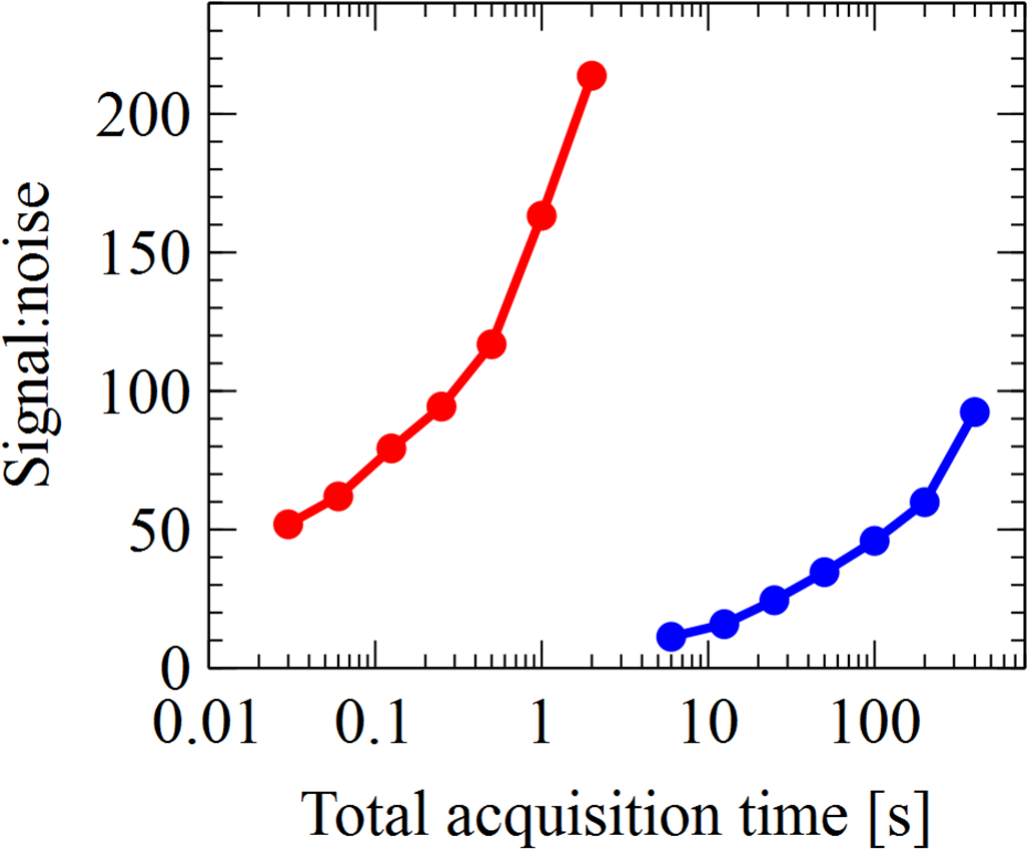
Signal to noise. A series of three 77 K emission spectra were acquired for each different integration time from both the table-top fluorometer (blue) and the custom fluorometer (red), using the cyanobacterium *Synechocystis*. The integration time is representative of the full spectrum acquisition (200 nm scan for the table-top fluorometer). The set of average emission spectra from each fluorometer was divided by their average standard deviation as a proxy for signal to noise. The samples had an OD_750_ of 1.0, and chlorophyll concentration of 2.9 nmol • mL^-1^.

For many experiments, the acquisition time is a critical element. Fast data acquisition may not only be a matter of convenience but also allow novel types of measurements. We acquired data, while varying the acquisition time and evaluated the signal to noise characteristics for both fluorometers (Fig 6). The recorded spectra at different acquisition rates indicate that the constructed 77K fluorescence instrument is able to clearly resolve fluorescence spectra for *Synechocystis* at a cell density of 1 OD (a chlorophyll concentration of 2.9 nmol • mL^-1^) in 0.5 s. The comparable signal to noise characteristics in the traditional fluorometer would require an integration time of 2 s • nm^-1^, meaning an acquisition of a 200 nm spectrum would take 400 s, 800 times longer than the constructed fluorometer.

#### Phycobilin fluorescence

Fluorescence spectra at 77K that are the result of excitation of the phycobilin-containing antenna system provide additional information on the status of the photosynthetic machinery in cyanobacteria, rhodophytes and glaucophytes. We therefore evaluated the ability of the constructed instrument to acquire these fluorescence spectra.

**Fig 7:**
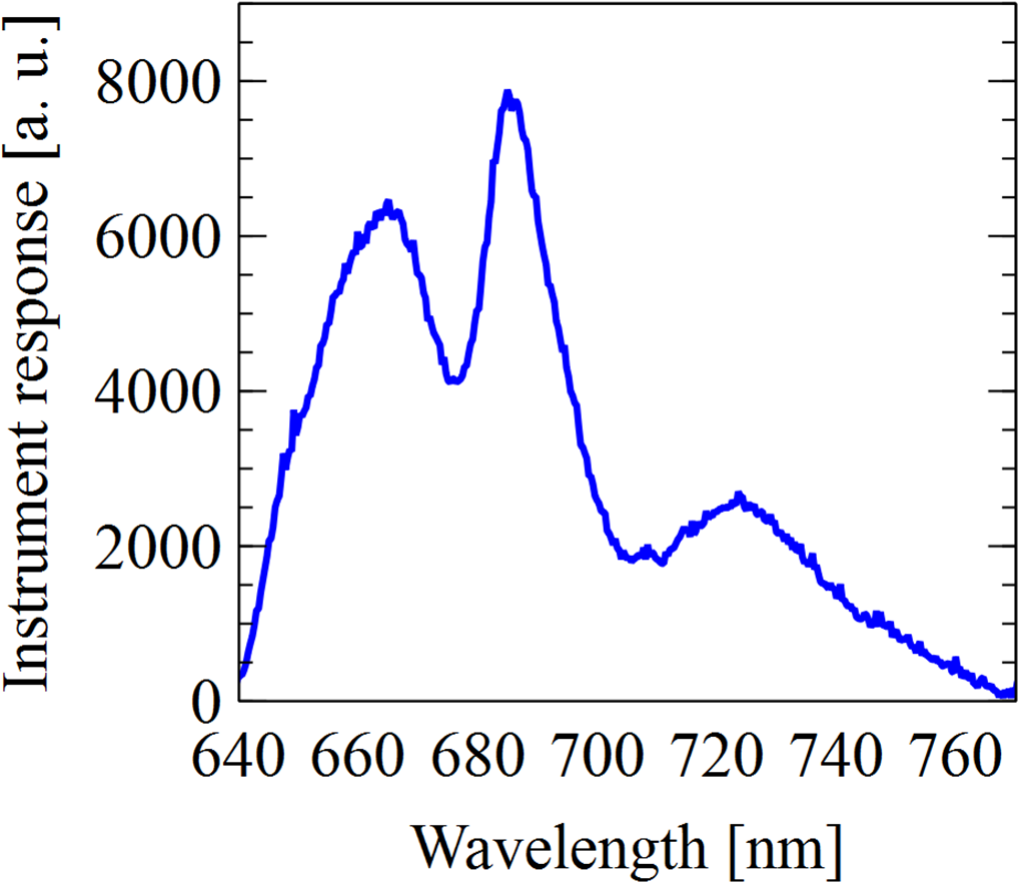
Alternative pigment excitation. Emission spectrum obtained from the custom fluorometer. *Synechocystis* cells were measured at a chlorophyll concentration of 2.9 nmol • mL(OD_750_ of 1) and frozen at 77 K. This sample was then excited with a 572 nm LED, targeting phycobilin proteins.

To acquire 77K fluorescence spectra resulting from phycobilin excitation, we used a 572 nm LED as excitation source in the custom setup. To prevent excitation light being detected by the OceanOptics spectrometer, a 580 nm long-pass filter was inserted between the dewar and the optical fiber that delivers the fluorescence emission to the CCD array spectrometer. Although the 580 nm long-pass filter prevented the majority of longer wavelength light emitted by the 572 nm LED to interact with the sample, the excitation light produced an observable shoulder in the emission spectra. To remove this shoulder a logarithmic decay function was subtracted from the raw emission spectra to obtain corrected emission spectra (Fig A in S1 File). The corrected 77K fluorescence emission spectrum of *Synechocystis* excited at 77K phycobilin excitation is presented in Fig 7.

## Discussion and Conclusions

We interfaced a CCD array detector with a LED-based illumination system and liquid nitrogen dewar to acquire fluorescence emission spectra at 77 K resulting from chlorophyll *a* and phycobilin excitation. This instrument was compared to a traditional table-top fluorometer using the cyanobacterium *Synechocystis*, and the green alga *Chlamydomonas as* examples.

Investigating signal to noise ratios during different acquisition times revealed that the custom-made fluorometer compares favorably to the traditional table-top fluorometer.

We determined the range cell concentrations that result in fluorescence spectra, which are undistorted by re-absorption events. Undistorted emission spectra between the chlorophyll concentration of 1.0–5.0 nmol•mL^-1^ for both *Synechocystis* and *Chlamydomonas* were observed. At higher chlorophyll concentrations, a small deviation in this linear relationship may occur due to reabsorption of emitted light. It is of note that the used sample tubes have an inner diameter of 2 mm and therefore offer a short path length. As the thickness of the frozen sample will change absorption parameters, fluorescence spectra should be evaluated at different chlorophyll concentrations when a system is established.

The constructed instrument enables the rapid and reliable assessment of 77 K fluorescence emissions in a compact and portable design, making it suited for 77 K fluorescence emission determination in the field. The constructed 77K fluorometer is based on an off-the shelf components with the CCD array fluorometer as the main expense (~$3300 US). Once parameters such as LED brightness and chlorophyll concentration have been determined, the setup delivers data reliably for long periods of time, as the CCD array spectrometer unit and LEDs are very stable over time. Regular checks with suitable standards are of course good scientific practice.

The presented methodology and instrumentation may also be adapted to determine 77 K fluorescence emission of other organisms. As the OceanOptics spectrometer has a spectral range that includes near infrared, 77K chlorophyll fluorescence spectra measurements of heliobacteria, green sulfur bacteria, filamentous anaerobic phototrophs and purple bacteria are possible with this instrument.

As shown with *Synechocystis*, an instrument with alternative LEDs for excitation is capable of exciting other light harvesting pigments and complexes within the organism. All that is seemingly required is to adapt the LED and long-pass filter combination to the spectral characteristics of the desired organisms.

In summary, the construction and performance parameters of a 77 K fluorometer has been presented. The straightforward calibration of the instrument, high temporal resolution, and high signal to noise ratios of this compact and portable instrument may create research opportunities in the laboratory and the field.

## Supporting Information

S1 File This file contains the following figure: Fluorescence emission baseline-subtraction (Fig A in S1 File). (DOC)
