# Supplementary material for: A Practical Solution for 77 K Fluorescence Measurements Based on LED Excitation and CCD Array Detector
Source: PLoS One. 2015 Jul 15;10(7):e0132258. doi: 10.1371/journal.pone.0132258 (PMC4503352; doi:10.1371/journal.pone.0132258)
Supplement: S1 File — (DOC) [file pone.0132258.s001.doc]

**S1 File**

A portable solution for 77 K fluorescence measurements based on LED excitation and CCD array detector

This document includes:

S1 Fig.A. Fluorescence emission baseline-subtraction

**
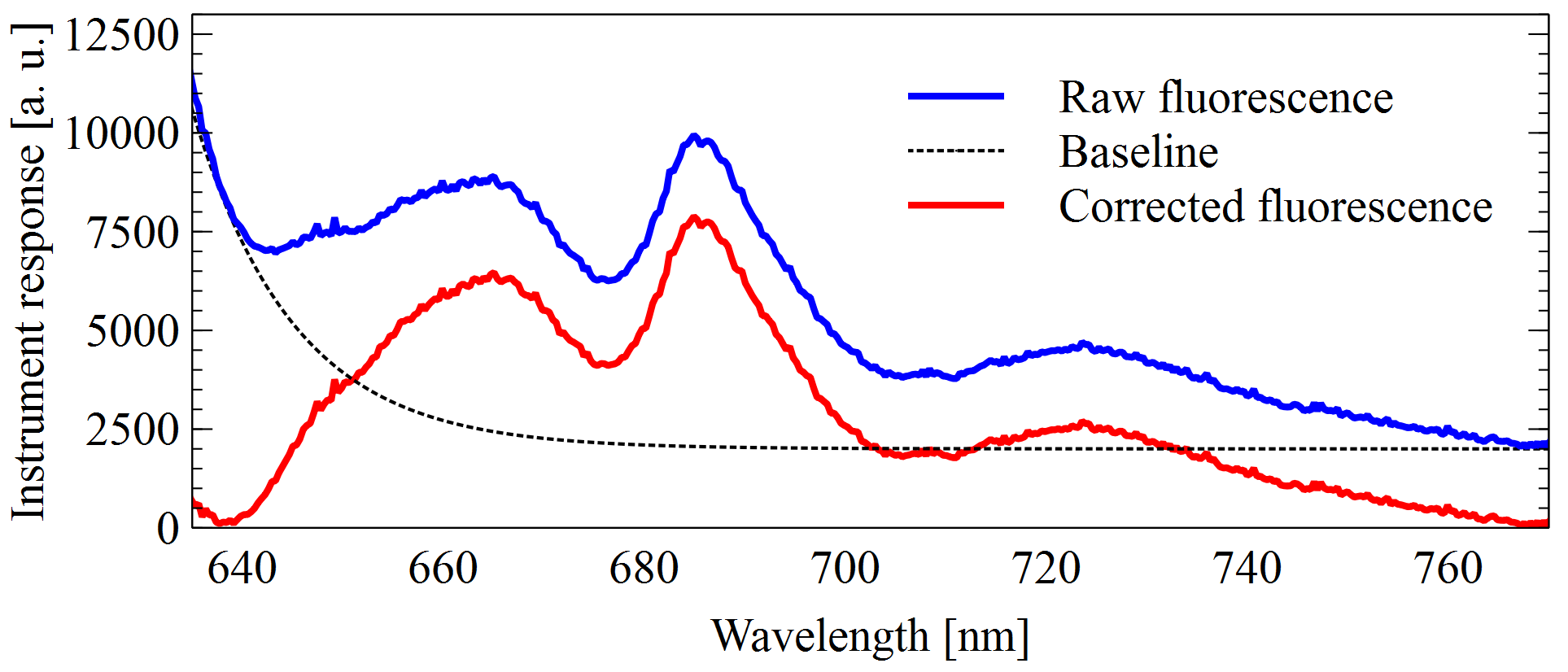
S1 Figure A. Fluorescence emission baseline-subtraction.** A logarithmic decay curve (dotted line) was modeled to the shoulder of the 572 nm LED light recorded in the fluorometer without a sample (a blank measurement). This modeled baseline is representative of the excitation LED shoulder, and is therefore subtracted from the raw emission spectrum (blue line) to obtain a corrected fluorescence emission spectrum (red line).
